# Constructing a new prognostic signature of gastric cancer based on multiple data sets

**DOI:** 10.1080/21655979.2021.1940030

**Published:** 2021-06-23

**Authors:** Liqiang Zhou, Hao Lu, Fei Zeng, Qi Zhou, Shihao Li, You Wu, Yiwu Yuan, Lin Xin

**Affiliations:** Department of General Surgery, The Second Affiliated Hospital of Nanchang University, Nanchang, Jiangxi, P.R China

**Keywords:** Gastric cancer, prognostic signature, bioinformatics, nomogram

## Abstract

In order to explore new prediction methods and key genes for gastric cancer. Firstly, we downloaded the 6 original sequencing data of gastric cancer on the Illumina HumanHT-12 platform from Array Expression and Gene Expression Omnibus, and used bioinformatics methods to identify 109 up-regulated genes and 271 down-regulated genes. Further, we performed univariate Cox regression analysis of prognostic-related genes, then used Lasso regression to remove collinearity, and finally used multivariate Cox regression to analyze independent prognostic genes (MT1M, AKR1C2, HEYL, KLK11, EEF1A2, MMP7, THBS1, KRT17, RPESP, CMTM4, UGT2B17, CGNL1, TNFRSF17, REG1A). Based on these, we constructed a prognostic risk proportion signature, and found that patients with high-risk gastric cancer have a high degree of malignancy. Subsequently, we used the GSE15459 data set to verify the signature. By calculating the area under the recipient operator characteristic curve of 5-year survival rate, the test set and verification set are 0.739 and 0.681, respectively, suggesting that the prognostic signature has a moderate prognostic ability. The nomogram is used to visualize the prognostic sig-nature, and the calibration curve verification showed that the prediction accuracy is higher. Finally, we verified the expression and prognosis of the hub gene, and suggested that HEYL, MMP7, THBS1, and KRT17 may be potential prognostic biomarkers.

## Graphical abstract

**Figure:**
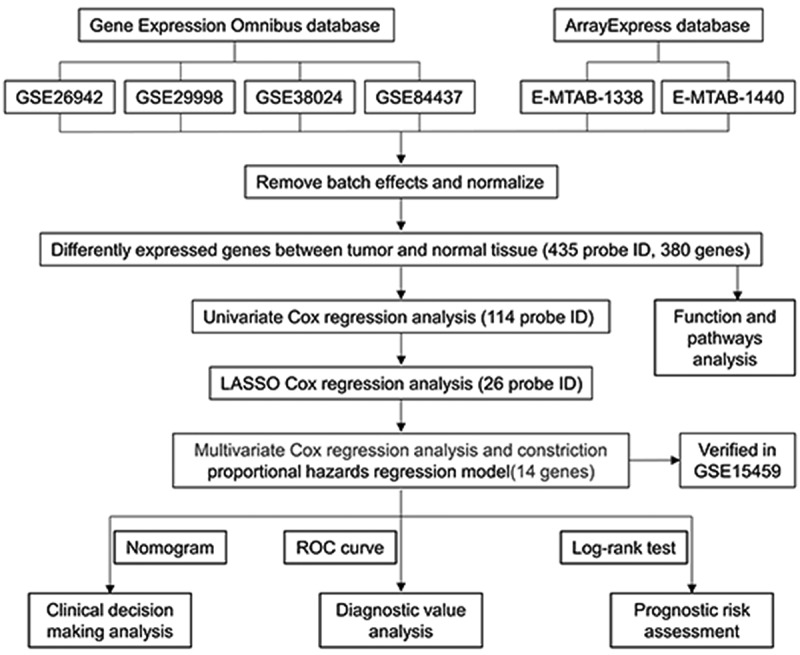

## Introduction

Gastric cancer (GC) is one of the most common tumors in the world, and many factors are involved in its pathogenesis, such as low fruit and vegetables, high salt intake, Epstein-Barr virus and Helicobacter pylori infection [1]. The latest statistics showed that globally, GC deaths reached 770,000, and new cases reached 1.09 million [2]. There are usually no obvious clinical symptoms in early gastric cancer, the patient was already with advanced gastric cancer at the time of treatment. In China, due to the extensive metastasis of advanced gastric cancer and strong drug resistance, the 5-year survival rate is less than 30%[3]. However, the diagnosis method of gastric cancer has the disadvantage of not being able to detect and diagnose early. Traditional molecular biomarkers such as CEA and CA199 lack specificity and cannot detect early gastric cancer. Although there are a variety of new diagnostic techniques and new treatment methods, they have not yet been widely used [4,5]. Therefore, it is great significant to find new and effective biomarkers on the basis of removing individual differences.

At present, sequencing technology can analyze the potential changes in the occurrence and development of diseases from the entire genome level, and has become one of the important means to find the mechanism of disease occurrence and development. However, due to the extensive heterogeneity of tumors, different patients show different specificities and prognosis. Therefore, if a large sample analysis can be used to obtain key candidate genes related to the prognosis of tumors, it will lay a solid foundation for tumor prevention and treatment. In addition, reports indicated that the combination of multiple prognostic factors is stronger than a single prognostic factor, and has more accurate performance [6,7].

With the development and improvement of public databases, the use of databases to mine prognostic genes and construct prognostic models for different tumors can accurately predict the survival of tumor patients, which is a promising method [8–10]. Compared with traditional tumor markers, gene-based prognostic models can provide more accurate prediction capabilities. The purpose of our research is to construct a hazard ratio model to predict the survival rate of gastric cancer patients. On this basis, we integrated and analyzed 6 types of gastric cancer sequencing chips on the same platform, and obtained 14 central genes through systematic statistical and bioinformatics methods to construct a prognostic signature and analyze them through external data sets. Since our prediction model directly reflects the progression of the tumor at the gene level, it can provide more accurate survival predictions for gastric cancer and more personalized treatment methods based on the gene level, which ultimately improve the survival rate of gastric cancer patients.

## Materials and methods

### Data collection and data processing

We downloaded Illumina HumanHT-12 V3 and Illumina HumanHT-12 V4.0 platform gastric cancer tissue microarray raw data from GEO (https://www.ncbi.nlm.nih.gov/gds/) and ArrayExpression (https://www.ebi.ac.uk/arrayexpress/), the microchip data set included GSE26942, GSE29998, GSE38042, GSE84437, E-MTAB-1338, E-MTAB-1440. We first used the ‘lumi’ R package to extract the expression-based data of each microarray [11]. Subsequently, the ‘sva’ R package normalized each microarray data, removed batch effects, and combined 6 microarray data for analysis [12]. Using the ‘limma’ R package to analyze differentially expressed genes for the combined data set [13], the screening threshold is |logFC|>1, *P* < 0.05, and the difference genes obtained were processed in the next step.

### Kyoto encyclopedia of genes and genomes (KEGG) and gene ontology (GO) enrichment analysis

GO annotation project has carried out a consistent description of gene functions, developed a controllable vocabulary, and has no species specificity. It includes Cellular Component (CC), Molecular Function (MF) and Biological Process (BP). KEGG is a comprehensive database that integrates information on genomes, chemistry, and system functions. We used the ‘org.Hs.eg.db’ R package to perform enrichment analysis on these differential genes, and set the filtering conditions to P < 0.05 and FDR<0.05 [14]. The obtained items are visualized using the ‘enrichplot’ and ‘ggplot2’ R packages. In order to systematically understand the potential pathways of these differential genes regulating gastric cancer.

### Construction of prognosis model

In order to screen out the differential genes related to overall survival, use the ‘survival analysis’ package to perform univariate Cox regression analysis, and screen out prognostic-related genes with *P* < 0.05. In addition, we used Lasso regression analysis to screen out genes related to the prognosis of gastric cancer that are more significantly related to the prognosis of gastric cancer. According to the results of lasso regression analysis, the multivariate Cox regression analysis was used to find the independent predictive gastric cancer hub gene and construct a risk ratio model, respectively, to calculate the HR value, 95% confidence interval of HR and the corresponding regression coefficient (β). We calculated the risk score of each gastric cancer patient based on the model and the hub gene expression level (Exp), the formula is as follows: Risk = β1*Exp1+ β2*Exp2+ βi*Expi.

Therefore, gastric cancer patients were divided into high-risk groups and low-risk groups. Kaplan-Meier was used to draw survival curves and Log-Rank test was used to analyze the difference in OS between the two groups of patients. The ‘Survival ROC’ package was used to draw a 5-year ROC curve and calculate the AUC value to evaluate the predictive ability of the predictive model [15]. Univariate and multiple Cox regression analysis were used to evaluate the risk score and clinicopathological characteristics of HR and P values to determine independent prognostic factors for patients with gastric cancer. In addition, we also use the Chi-square test to analyze the relationship between high and low risk and the expression of each gene and each clinicopathological feature.

### Gene set enrichment analyses (GSEA)

GSEA can fully enrich the biological differences between samples of different classifications as a whole^[16]^. In this study, GSEA4.0 based on the molecular signature database (MSigDB) was used, hallmark7.1 was used as the comparison gene set, and the Number of permutations was set to 1000 for enrichment. Use NES>1 and FDR<0.001 as the screening conditions to identify the similarities and differences between the occurrence and development of gastric cancer in the high-risk group and the low-risk group.

### Construction and verification of nomogram

We drew a nomogram according to the prognostic risk model, obtained the corresponding score by analyzing the hub gene expression level, and added the points of all hub genes to obtain the corresponding total points [17]. By drawing a vertical line, we can predict the probability of survival for patients with gastric cancer in 1 to 5 years. In addition, in order to test the prediction ability of the nomogram, we draw a 5-year calibration curve by analyzing the survival probabilities of the predicted value and the actual value at the quartile of all gastric cancer patients. If the actual value is close to the predicted value, the nomogram has good predictive performance.

### Hub gene expression and prognostic verification

The human protein atlas (HPA, https://www.proteinatlas.org/) database contains the tissue and cell distribution information of 24,000 human proteins [18]. This database was used to verify the hub genes. Kaplan-Meier Plotter website (http://kmplot.com/) includes the expression and survival information of the gastric cancer GPL570 platform chip in the GEO database, which can quickly analyze the relationship between genes and overall prognosis^[19]^. We use this tool to verify the prognostic ability of hub genes.

## Results

Gastric cancer is a common disease with high mortality worldwide. The clinical symptoms of early gastric cancer are not obvious, resulting in poor prognosis and high recurrence rate. Therefore, some technical means are needed to improve the accuracy of diagnosis and prognosis. Because gastric cancer has a high degree of heterogeneity, it is more suitable to study gastric cancer at the genetic level. Through the analysis of the database, we have obtained 14 core genes to construct the prediction model of gastric cancer, and the verification of external data showed that it has good predictive performance. We finally constructed a nomogram to visually represent the survival rate of 1–5 years, which helps individualized and precise treatment. For the obtained hub gene, the expression and prognosis were verified in the HPA database and Kaplan-Meier Plotter. It was finally confirmed that HEYL, MMP7, THBS1, and KRT17 may be potential biomarkers of gastric cancer.

### Identify differentially expressed genes (DEGs)

**Figure 1.**
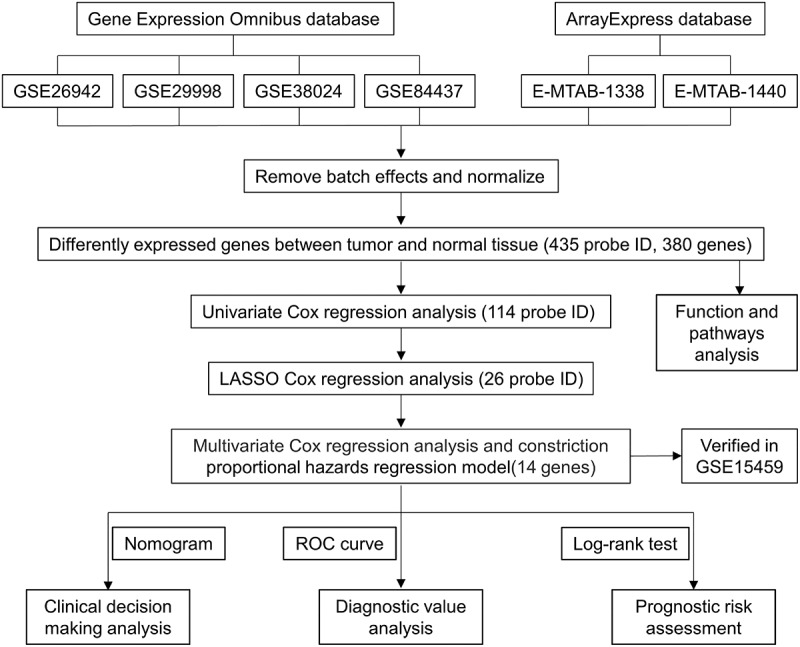
Flow chart of this research

**Figure 2.**
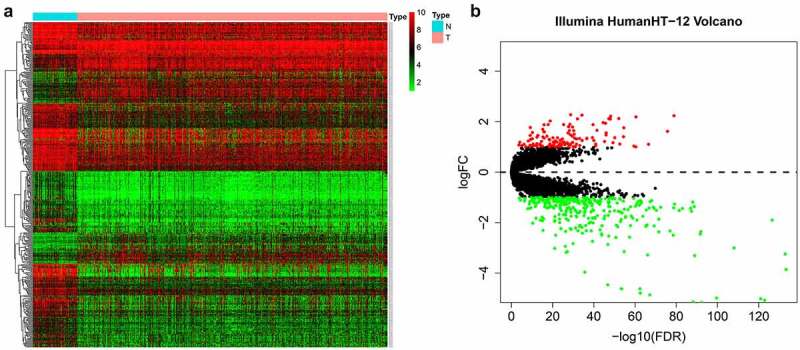
The differentially expressed genes in gastric cancer

Our entire research process has been shown in Figure 1. We downloaded 4 data sets (GSE26942, GSE29998, GSE38024, GSE84437) from the GEO database and 2 data sets (E-MTAB-1338, E-MTAB-1440) on ArrayExpress. We extracted the original expression data and removed the batch effect. After standardization, we obtained a total of 118 normal gastric tissue samples and 827 gastric cancer samples. After using the ‘limma’ package analysis, a total of 380 genes meets the screening conditions, of which 109 genes are up-regulated, and 271 genes are down-regulated **Table S1**). We used R to draw heat maps and volcano maps of these differential genes (Figure 2(a, b)).

(A) Heat map; (B) Volcano plot.

### GO and KEGG function enrichment analysis

**Figure 3.**
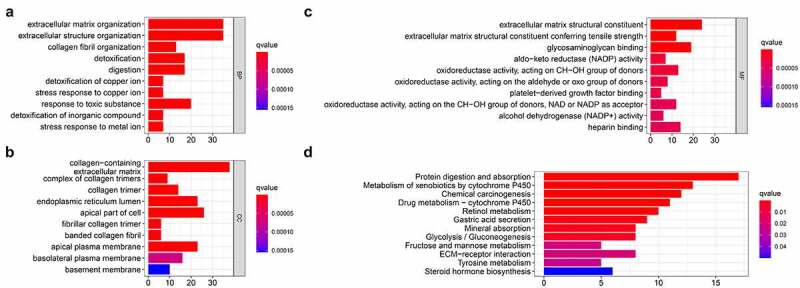
Functional enrichment analysis of the DEGs

In order to study the mechanism of differential genes affecting gastric cancer, we used GO and KEGG to further analyze the potential mechanisms of these DEGs in regulating gastric cancer. Among them, GO enrichment analysis showed that BP is enriched in extracellular matrix organization, extracellular structure organization, collagen fibril organization, detoxification, digestion, detoxification of copper ion, stress response to copper ion, response to toxic substance, detoxification of inorganic compound, stress response to metal ion; CC is enriched in collagen-containing extracellular matrix, complex of collagen trimers, collagen trimer, endoplasmic reticulum lumen, apical part of cell fibrillar collagen trimer, banded collagen fibril, apical plasma membrane, basolateral plasma membrane, basement membrane; MF is enriched in extracellular matrix structural constituent, extracellular matrix structural constituent conferring tensile strength, glycosaminoglycan binding, aldo-keto reductase (NADP) activity, oxidoreductase activity, platelet-derived growth factor binding, oxidoreductase activity, alcohol dehydrogenase (NADP+) activity, heparin binding (Figure 3(a-c)). The results of KEGG indicated that the DEGs are enriched in Chemical carcinogenesis, Glycolysis/Gluconeogenesis, ECM-receptor interaction, etc (Figure 3d).

(A) The biological process enrichment results of GO with different genes. (B) The cellular components enrichment results of GO with different genes. (C) The molecular function enrichment results of GO with different genes. (D) KEGG enrichment results of differential genes.

### Identify prognostic-related hub genes

**Figure 4.**
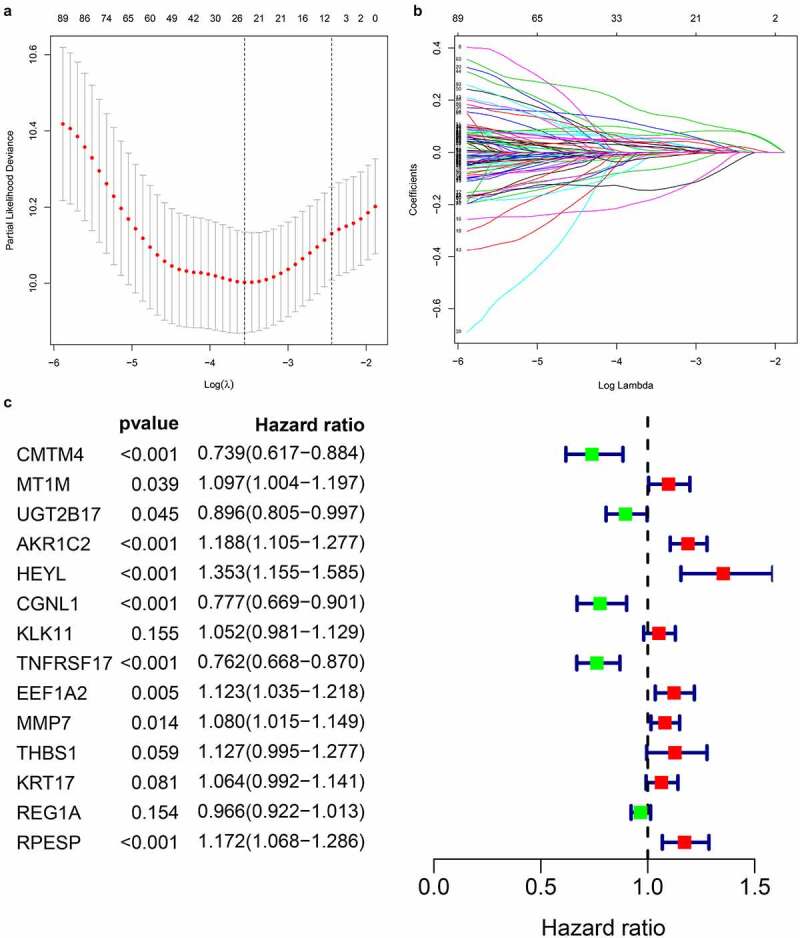
Prognosis-related gene screening

By extracting clinical data from each data set, we obtained a total of 635 gastric cancer samples with clinical data. Using univariate Cox regression to analyze the relationship between 435 differential gene probes and prognosis, 114 probes related to prognosis were subsequently obtained, of which 77 probes with HR>1 and 37 probes with HR<1. In addition, we used lasso regression to perform further 114 prognostic-related probes, and we obtained 26 gene probes significantly related to prognosis (Figure 4(a, b)). We also used multivariate Cox regression analysis to test the results obtained in the previous step to find independent prognostic factors among the 26 probe IDs. The results showed (Figure 4c) that a total of 14 hub genes are independent prognostic factors for gastric cancer patients. Among them, those with HR>1 include MT1M, AKR1C2, HEYL, KLK11, EEF1A2, MMP7, THBS1, KRT17, RPESP, HR< 1 Including CMTM4, UGT2B17, CGNL1, TNFRSF17, REG1A.

(A, B) Selecting the best parameters for gastric cancer in LASSO regression analysis, and 26 gene probes significantly related to prognosis; (C) Multivariate Cox regression analysis to get prognosis related 14 genes.

### Prognostic model construction and verification

Based on multivariate Cox regression, we constructed a 14-gene risk ratio model to predict the prognosis of gastric cancer patients. By assigning the corresponding regression coefficient to each gene, the risk-score of each gastric cancer patient can be calculated. The risk score formula of this model is as follows:

*Risk score *= (−0.303 * ExpCMTM4) + (0.092 * ExpMT1M)+ (−0.110 * ExpUGT2B17) + (0.172 * ExpAKR1C2)+ (0.302 * ExpHEYL)+ (−0.253 * ExpCGNL1)+ (−0.051 * ExpKLK11)+ (−0.272 * ExpTNFRSF17)+ (−0.116 * ExpEEF1A2)+ (−0.077 * ExpMMP7)+ (- 0.120 * ExpTHBS1)+ (−0.062 * ExpKRT17)+ (−0.034 * ExpREG1A)+ (−0.159 * ExpRPESP).

**Figure 5.**
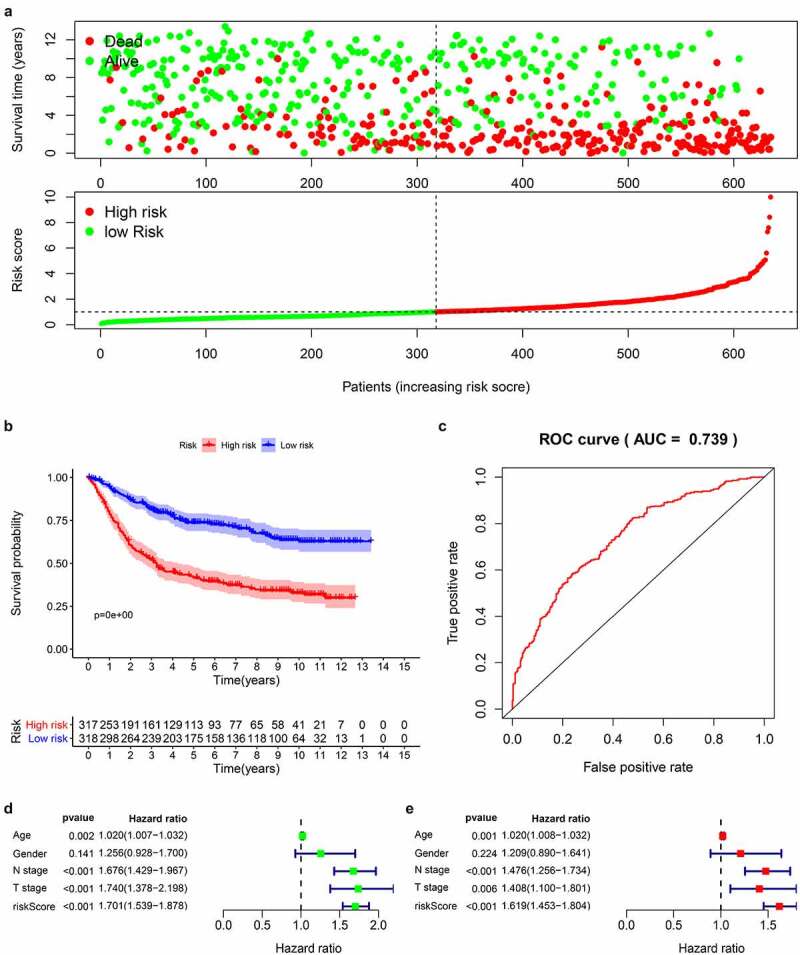
Prognostic analysis of 14-genes signature in the train cohort

According to the obtained risk scores, the median is divided into high and low risk groups. The dot plot of survival status revealed (Figure 5a) that the number of deaths of gastric cancer patients in the high-risk group is greater and their survival time is less than that in the low-risk group. We drew the K-M curve (Figure 5b) and used log-Rank to test. The results suggested that the high-risk group has a worse survival rate than the low-risk group (P < 0.05). Then, we drew a 5-year ROC curve to judge the predictive ability of the prognostic signature, and calculated the AUC to be 0.739, showed that our prediction model has a medium ability to predict (Figure 5c). In addition, we also combined the clinicopathological characteristics to analyze whether the prognostic signature is an independent prognostic factor. Univariate Cox regression analysis showed Age [HR = 1.020, 95%CI (1.007–1.032)), P = 0.002], N stage [HR = 1.676, 95%CI (1.429–1.967), P < 0.001], T stage [HR = 1.740, 95%CI (1.378–2.198), P < 0.001], risk score [HR = 1.700, 95%CI (1.539–1.878), P < 0.001] are related to prognosis (Figure 5d). Multivariate Cox regression analysis showed Age [HR = 1.020, 95%CI (1.008–1.032), P = 0.001], N stage [HR = 1.476, 95%CI (1.256–1.734), P < 0.001], T stage [HR = 1.408, 95%CI (1.100–1.801), P = 0.006], risk score [HR = 1.619, 95%CI (1.453–1.804), P < 0.001] are independent prognostic factors for gastric cancer patients and showed that the risk score has the best predictive ability (Figure 5e).

(A) Scatterplots of GC patients with different survival status in training group; Risk score distribution of GC patients with different risks (low, green; high, red) in the testing group; (B) Kaplan–Meier Survival curve of low-risk and high-risk subgroups; (C) 5-year time-dependent ROC for survival prediction models; (D) Univariate Cox regression analysis on the prognosis of clinicopathological characteristics and risk scores in patients with GC; (E) Multivariate Cox regression analysis on the prognosis of clinicopathological characteristics and risk scores in patients with GC.

**Figure 6.**
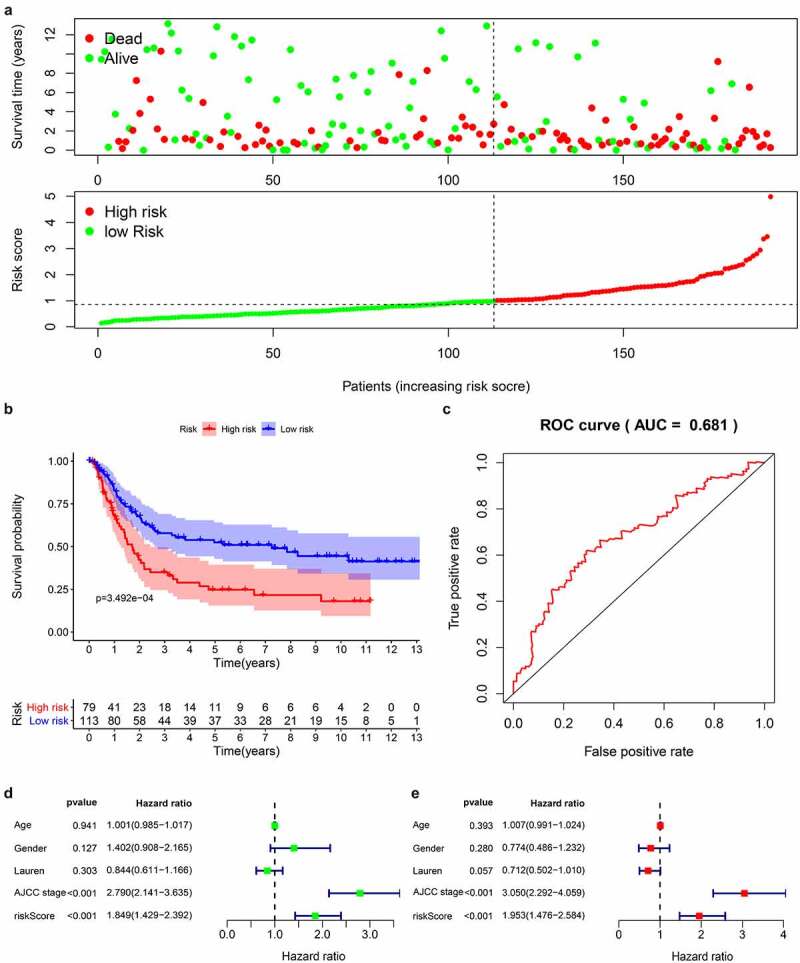
Prognostic analysis of 14 genes signature in the GSE15459 data set

We downloaded the original data and clinical information of the GSE15459 dataset from the GEO database and used the RMA algorithm to extract the expression of each gene to verify the 14-gene prediction model. The survival status chart (Figure 6a) showed that the high-risk group (n = 79) has a higher number of deaths and a lower survival time than the low-risk group (n = 113). The K-M curve (Figure 6b) confirmed that the high-risk group has a lower survival rate than the first-risk group. We also drew a 5-year ROC curve, and the AUC of the GS415459 cohort is 0.681 (Figure 6c). Combined with clinical case parameters, univariate Cox regression analysis indicated that AJCC stage [HR = 2.790, 95%CI (2.141–3.635), P < 0.001] and risk score [HR = 1.849, 95%CI (1.429–2.392), P < 0.001] has prognostic ability (Figure 6d). Multivariate Cox regression analysis showed that AJCC stage [HR = 3.050, 95%CI (2.292–4.059), P < 0.001] and risk score [HR = 1.953, 95%CI (1.476–2.584), P = 0.006] are independent prognostic factors (Figure 6e). The results of the analysis in the validation data set are similar with the model we built.

(A) Scatterplots of GC patients with different survival status in training group; Risk score distribution of GC patients with different risks (low, green; high, red) in the testing group; (B) Kaplan–Meier Survival curve of low-risk and high-risk subgroups; (C) 5-year time-dependent ROC for survival prediction models; (D) Univariate Cox regression analysis on the prognosis of clinicopathological characteristics and risk scores in patients with GC; (E) Multivariate Cox regression analysis on the prognosis of clinicopathological characteristics and risk scores in patients with GC.

### Bioinformatics analysis

**Figure 7.**
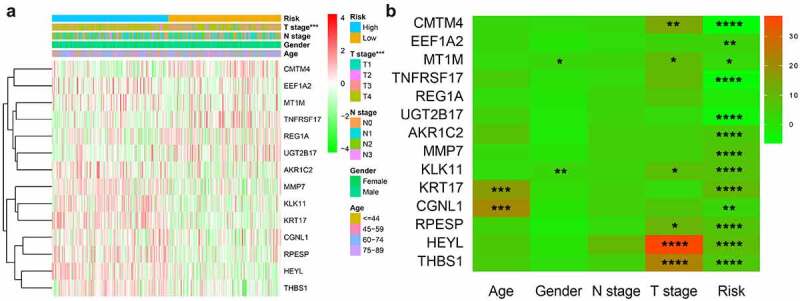
Risk and clinicopathological characteristics of 14 genes

**Figure 8.**
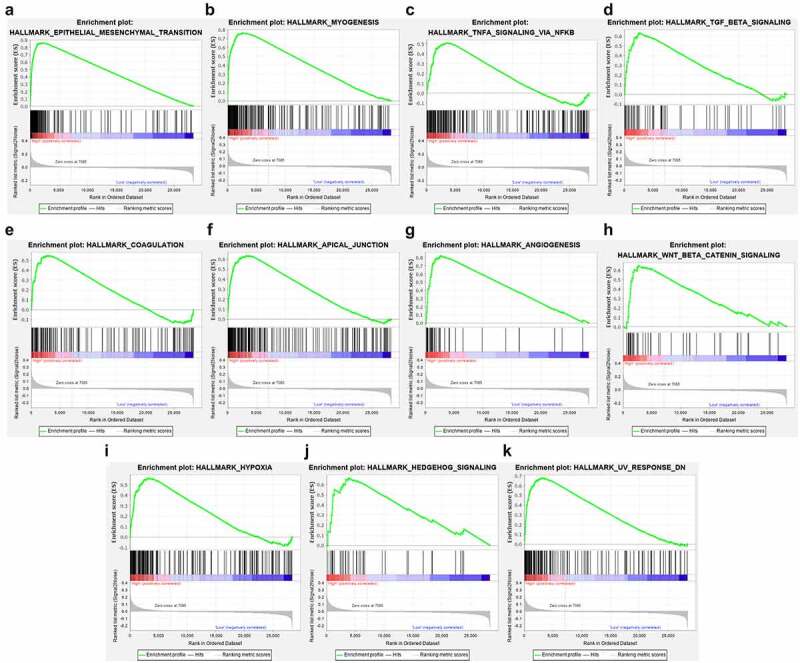
High-risk group conducts GSEA enrichment pathway analysis

We used chi-square test to explore whether there is a relationship between each clinicopathological feature and high and low risk groups. The results showed that the risk grouping is related to T stage (Figure 7a). The high-risk group gastric cancer patients were mostly in T3 and T4 stages, and more deaths. We also analyzed the relationship between 14 hub genes and clinicopathological characteristics. Analysis suggested (Figure 7b) that almost all hub genes are related to risk scores. In addition, CMTM4, MT1M, KLK11, RPESP, HEYL, THBS1 are related to T stage. To identify the difference between high and low risk groups, we used GSEA for analysis. The enrichment indicated that the samples of the high-risk group were enriched in Epithelial Mesenchymal Transition, Myogenesis, NF-KB/TNF-α via, TGF-β signal, Goagulation, Apical junction, Angiogenesis, Hypoxia, Hedgehog signal, UV response down (Figure 8).

(A) The relationship between clinicopathological characteristics and risk group; (B) The relationship between 14 genes expression level and clinicopathological characteristics and risk value.

### Nomogram construction and verification

**Figure 9.**
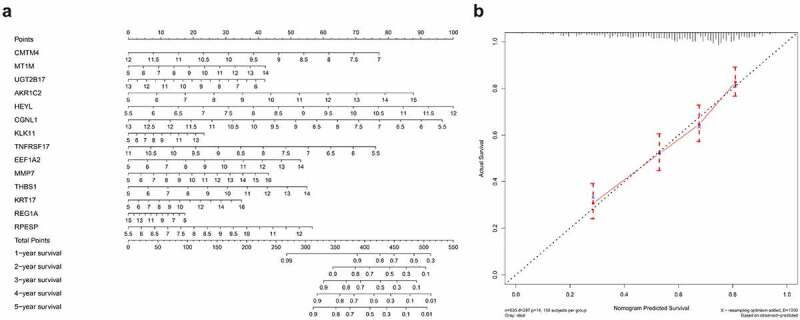
Establishment and validation of Nomogram (a) Nomogram for predicting 1–5 years OS of GC patients. (b) calibration chart for nomogram accuracy

Based on the constructed 14-gene prognostic model, we drew nomograms for better helping clinicians to make precise treatment decisions, thereby improving the survival time and quality of life of patients with gastric cancer. By detecting the expression levels of 14 hub genes and assigning corresponding scores, the 1–5 years survival rate of gastric cancer patients can be judged after adding the total scores (Figure 9a). In order to test the predictive ability of the nomogram, we tested the actual survival rate and predicted survival rate of 635 gastric cancer samples respectively and drew a 5-year calibration curve. The results showed that the calibration curve is almost in line with the standard line, suggesting that our connection diagram has very accurate capabilities (Figure 9b).

### Hub genes expression and prognostic verification

**Figure 10.**
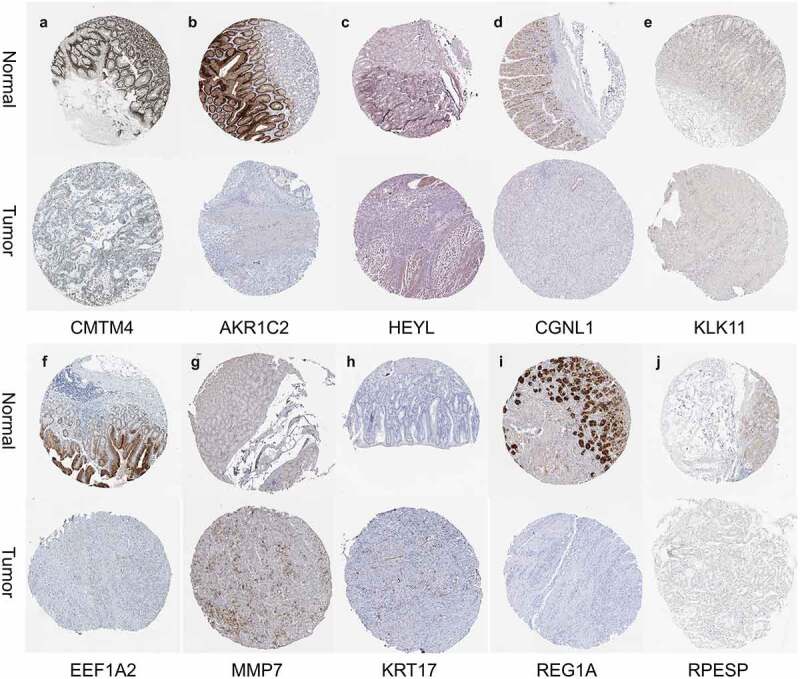
Verification of 14 genes expression in GC and normal gastric tissue using the HPA database

**Figure 11.**
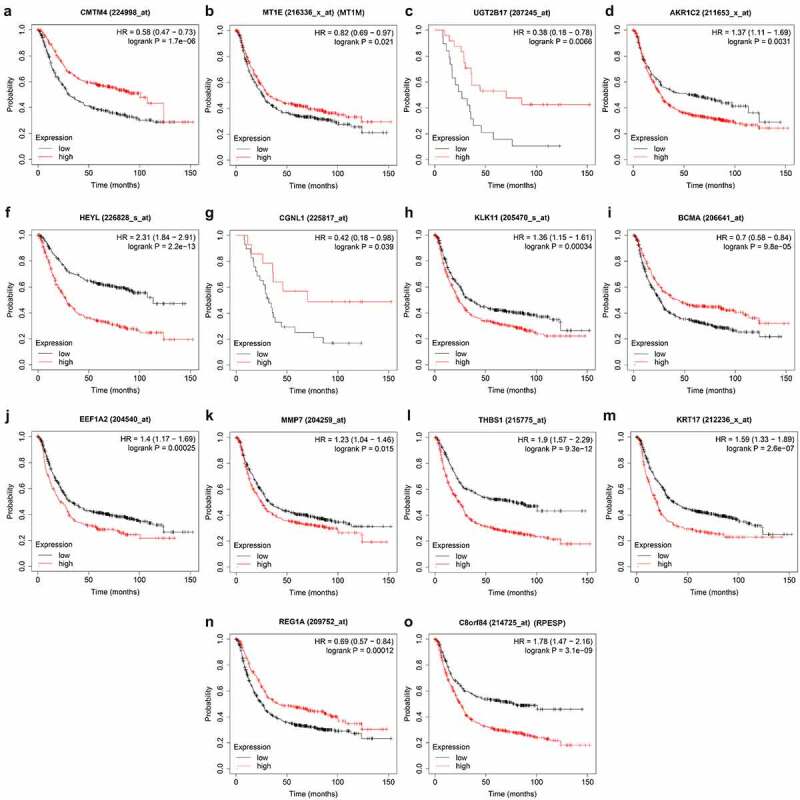
Validation the prognostic value of 14 genes in GC by Kaplan Meier-plotter

First, we searched the HPA database for the immunohistochemical data of CMTM4, AKR1C2, HEYL, CGNL1, KLK11, EEF1A2, MMP7, KRT17, REG1A, RPESP, and indicated that they were consistent with the mRNA level expression we analyzed (Figure 10). Secondly, for prognostic verification of the 14 hub genes, we used the Kaplan-Meier Plotter website. The results showed (Figure 11) that AKR1C2, HEYL, KLK11, EEF1A2, MMP7, THBS1, KRT17, C8ORF84 are associated with poor prognosis. The expression of CMTM4, MT1E, UGT2B17, CGNL1, BCMA, and REG1A are associated with better prognosis. In summary, HEYL, MMP7, THBS1, and KRT17 are not only highly expressed in gastric cancer, but also associated with poor prognosis and may be potential prognostic biomarkers.

## Discussion

Gastric cancer is a highly heterogeneous malignant tumor. According to research, about 10% of gastric cancer patients showed familial aggregation, and 1–3% of gastric cancer patients will have germline mutations [20], Prognosis and treatment should be judged and selected from the genetic level. Gastric cancer of the same pathological type and stage could show different prognosis in different cases. Individualized treatment of gastric cancer patients at the genetic level also reflects the concept of precision medicine and improve the quality of life of patients. For example, HER2 oncogene amplification and HER2 protein overexpression occur in approximately 17–20% of gastric cancer patients, and it is more common in intestinal gastric cancer and cancers in the proximal stomach or gastroesophageal junction [21], according to HER2 gene Positive expression, the use of trastuzumab prolonged the survival rate of patients [22,23]. All the above indicated that due to the genetic instability and extensive heterogeneity of tumors, a single factor cannot accurately predict the occurrence, development and prognosis of gastric cancer.

In this study, we systematically analyzed the differential genes between gastric cancer and normal tissues, and obtained key genes after screening. GO enrich the above genes to show that they are mainly enriched in extracellular matrix organization, collagen fibril organization, etc. which means that the above genes are inseparable from the microenvironment of the tumor. Recent studies have shown that the tumor microenvironment not only significantly affects tumor growth, angiogenesis, chemotherapy resistance and immune regulation, but also plays an important role in tumor cell immune regulation, chemotherapy resistance, and recurrence and metastasis. It is also the target of emerging tumor-targeted therapeutic drugs [24]. KEGG is mainly enriched in Chemical carcinogenesis, Glycolysis/Gluconeogenesis, ECM-receptor interaction, etc. Glycolysis in tumor cells is the main way to obtain energy, which can promote the proliferation and metastasis of tumor cells [25]. The treatment of energy metabolism of tumors is also an important option. Such as, Wei et al. used the compound DT-13 to inhibit the expression of glucose transporter 1, thereby inhibiting glucose absorption and aerobic glycolysis, thereby inhibiting the proliferation of colon cancer [26].

We used systematic statistical analysis to identify independent prognostic genes and constructed a risk ratio model. According to the expression levels and statistical regression coefficients of hub genes, they are divided into low and high-risk groups. Among them, gastric cancer patients in the high-risk group have a lower survival rate and are closely related to the T stage. Combining clinicopathological information, using univariate and multivariate Cox regression analysis, the prognostic model and N stage and T stage are independent prognostic factors for patients with gastric cancer. In addition, we also calculated the 5 sticky AUC value, showing medium predictive power. The external data set GSE15459 also showed that the model has good predictive ability. Based on the prognostic signature, we also constructed a nomogram and verified its prediction accuracy with a calibration curve, indicating that it has good prediction performance.

In order to analyze the potential pathogenic mechanism of high-risk patients with gastric cancer, we conducted pathway analysis on the high-risk and low-risk groups through GSEA, and found that the high-risk group was mainly enriched in epithelial-mesenchymal transition (EMT), TGF-β, Wnt /β-catenin, NF-KB/TNF-α signals, etc. These signals are closely related to tumor progression. Studies have shown that abnormal activation of the Wnt /β-catenin signal pathway plays a vital role in the occurrence and metastasis of gastric cancer [27], and the activated Wnt /β-catenin signal pathway has been found in more than 30% of gastric cancers [28], therefore, Wnt/β-catenin signal may be a potential strategy for targeted therapy of gastric cancer. TGF-β signal transduction is a very important regulator in the human body. It can mainly regulate the growth of tissues and maintain the homeostasis of the internal environment. When this signal is out of regulation, it often leads to a series of diseases including cancer[29]. Studies in breast, lung, and pancreatic cancer have proved that TGF-beta plays a key role [30], and its mechanism may be to induce EMT to promote tumor growth and invasion. In this study, we found that multiple enrichment pathways in the high-risk group are closely related to EMT. Therefore, we speculated that the EMT pathway in gastric cancer is the confluence point of other genes and pathways and plays a vital role in the occurrence and development of the disease. In our study, the EMT pathway is also an obvious enrichment pathway, and the possible mechanism is the N6-methyladenosine modification mediated by METTL3, which regulates the effects of E-cadherin and non-coding RNA. [31,32].

Our prognostic model includes 14 genes including MT1M, AKR1C2, HEYL, KLK11, EEF1A2, MMP7, THBS1, KRT17, RPESP, CMTM4, UGT2B17, CGNL1, TNFRSF17, REG1A. Among them, HEYL, MMP7, THBS1, and KRT17 are not only highly expressed in gastric cancer, but are also independent prognostic risk factors for gastric cancer. HEYL is a member of the division-related family of transcription factors. It not only regulates the differentiation, self-renewal and proliferation of cancer cells, but also promotes tumor angiogenesis, so it plays an important role in tumor progression [33–35]. Studies have shown that the HEYL gene is significantly increased in patients with gastric cancer, usually showing a poor prognosis [36]. The mode of action of HEYL on gastric cancer has not yet been clearly studied, and further proof is needed in the future. MMP7, also known as stroma lysin, is a unique member of the matrix metalloproteinase family and plays a key role in the middle of the family. It is mainly expressed by tumor cells and is different from other family members, which is an important feature of it. Reports showed that the expression of MMP7 is associated with the poor prognosis of gastric cancer and is involved in the epithelial-mesenchymal transition of the tumor to induce metastasis [37]. The mechanism may be that MMP-7 can cleave E-cadherin in gastric cancer cells [38]. THBS1 is a secreted protein. Many studies have confirmed that it is highly expressed in gastric cancer stroma and is closely related to tumor growth and metastasis [39,40]. However, there are also reports showed that THBS1 is abnormally elevated in gastric cancer tissues, which is associated with a poor prognosis and enhances angiogenesis in gastric cancer cells [41–43]. KRT17 has been confirmed to be involved in the progression of a variety of tumors. In gastric cancer studies, it has been shown that KRT17 is closely related to tumor size, depth of invasion, lymph node metastasis, stage of tumor lymph node metastasis, vascular invasion and poor prognosis [44]. After inhibiting gastric cancer cell KRT17, it inhibits proliferation and metastasis and induces apoptosis, and its mechanism of promoting tumor progression may be mainly regulated by AKT/mTOR signal [44,45].

## Conclusion

In general, we used bioinformatics methods to identify differential genes and independent prognostic genes in gastric cancer. A prognostic signature is constructed on this basis, and after multiple verifications, it reflects good predictive performance. Furthermore, we have also established a nomogram, which can effectively predict the survival rate of gastric cancer patients and will help clinicians to make accurate judgments. Although our research has problems such as lack of clinical information. However, the shortcomings are not concealed. A huge number of samples are included in the revised prognosis model, which has wide applicability.

## Supplementary Material

Supplemental Material

## Highlights

- 380 differential genes were screened from 118 normal and 827 gastric cancer samples.
- Fourteen independent prognostic genes in gastric cancer were identified, and a prognostic signature was constructed with good predictive performance.
- Constructed a nomogram for forecasting 1-5 years, and verified its accuracy in predicting survival.
- HEYL, MMP7, THBS1, KRT17 may be potential biomarkers of gastric cancer.
