# Supplementary material for: Constructing a new prognostic signature of gastric cancer based on multiple data sets
Source: Bioengineered. 2021 Jun 23;12(1):2820–35. doi: 10.1080/21655979.2021.1940030 (PMC8806649; doi:10.1080/21655979.2021.1940030)
Supplement: Supplemental Material [file KBIE_A_1940030_SM3504.docx]

**supplement table**

Differentially expressed genes

| gene ID | arry ID | logFC | P.Value | adj.P.Val |
| --- | --- | --- | --- | --- |
| CST1 | ILMN_1753449 | 2.27 | 1.83E-31 | 1.86E-29 |
| IL8 | ILMN_2184373 | 2.26 | 3.93E-37 | 5.66E-35 |
| BGN | ILMN_2206746 | 2.23 | 6.47E-83 | 1.27E-79 |
| COL1A1 | ILMN_1701308 | 2.22 | 1.97E-51 | 6.98E-49 |
| COL1A2 | ILMN_2104356 | 2.19 | 5.29E-64 | 3.71E-61 |
| CLDN1 | ILMN_1724686 | 2.13 | 1.81E-55 | 7.65E-53 |
| SULF1 | ILMN_1702363 | 2.11 | 1.53E-50 | 5.11E-48 |
| SPP1 | ILMN_1651354 | 2.11 | 6.41E-35 | 8.11E-33 |
| THBS2 | ILMN_1678842 | 2.1 | 2.96E-51 | 1.03E-48 |
| MMP7 | ILMN_2192072 | 2.03 | 3.18E-21 | 1.49E-19 |
| COL8A1 | ILMN_1685433 | 1.96 | 9.71E-48 | 2.71E-45 |
| CTHRC1 | ILMN_1725090 | 1.93 | 7.24E-49 | 2.11E-46 |
| SPP1 | ILMN_2374449 | 1.9 | 3.87E-31 | 3.81E-29 |
| IL8 | ILMN_1666733 | 1.89 | 5.55E-28 | 4.35E-26 |
| MMP7 | ILMN_1685403 | 1.88 | 3.31E-20 | 1.41E-18 |
| CLDN7 | ILMN_2143685 | 1.88 | 1.25E-42 | 2.51E-40 |
| CEACAM6 | ILMN_1712522 | 1.88 | 3.74E-18 | 1.31E-16 |
| SFRP4 | ILMN_1810172 | 1.8 | 1.94E-30 | 1.84E-28 |
| MMP11 | ILMN_1655915 | 1.79 | 2.09E-32 | 2.24E-30 |
| OLFM4 | ILMN_2116877 | 1.77 | 4.48E-11 | 7.15E-10 |
| THY1 | ILMN_1779875 | 1.76 | 5.53E-58 | 2.86E-55 |
| MSLN | ILMN_2353161 | 1.7 | 1.99E-17 | 6.46E-16 |
| COL6A3 | ILMN_2307861 | 1.66 | 1.25E-44 | 2.91E-42 |
| KIAA1199 | ILMN_1813704 | 1.65 | 7.40E-31 | 7.21E-29 |
| FNDC1 | ILMN_1734653 | 1.65 | 5.28E-33 | 5.89E-31 |
| TIMP1 | ILMN_1711566 | 1.62 | 1.03E-79 | 1.68E-76 |
| H19 | ILMN_2148527 | 1.6 | 5.18E-18 | 1.78E-16 |
| KRT17 | ILMN_1666845 | 1.59 | 2.89E-18 | 1.02E-16 |
| PI3 | ILMN_1693192 | 1.57 | 3.54E-12 | 6.55E-11 |
| COL6A3 | ILMN_1706643 | 1.56 | 3.52E-49 | 1.06E-46 |
| RARRES1 | ILMN_1800091 | 1.55 | 3.64E-30 | 3.39E-28 |
| APOC1 | ILMN_1789007 | 1.5 | 3.24E-33 | 3.64E-31 |
| COL1A2 | ILMN_1785272 | 1.48 | 2.36E-31 | 2.37E-29 |
| COL5A2 | ILMN_1729117 | 1.48 | 3.87E-49 | 1.15E-46 |
| HS.579631 | ILMN_1881909 | 1.46 | 5.43E-15 | 1.38E-13 |
| CDH17 | ILMN_1814015 | 1.46 | 4.75E-11 | 7.54E-10 |
| CAMK2N1 | ILMN_1794863 | 1.43 | 2.79E-32 | 2.96E-30 |
| SERPINB5 | ILMN_1793888 | 1.43 | 1.39E-19 | 5.56E-18 |
| COL4A1 | ILMN_1653028 | 1.4 | 7.90E-58 | 4.03E-55 |
| UBD | ILMN_1678841 | 1.4 | 1.67E-17 | 5.46E-16 |
| KLK6 | ILMN_1780255 | 1.39 | 1.59E-14 | 3.84E-13 |
| TMEM158 | ILMN_1792455 | 1.37 | 2.47E-42 | 4.86E-40 |
| LOC730316 | ILMN_1662130 | 1.36 | 1.89E-53 | 7.37E-51 |
| IFI6 | ILMN_2347798 | 1.36 | 6.64E-25 | 4.14E-23 |
| FNDC1 | ILMN_2163873 | 1.36 | 1.29E-22 | 6.73E-21 |
| S100A9 | ILMN_1750974 | 1.35 | 3.87E-18 | 1.35E-16 |
| PRRX1 | ILMN_1739496 | 1.35 | 1.77E-33 | 2.06E-31 |
| COL12A1 | ILMN_1733756 | 1.34 | 1.47E-39 | 2.54E-37 |
| COL3A1 | ILMN_1773079 | 1.33 | 6.14E-36 | 8.24E-34 |
| FAP | ILMN_2232854 | 1.33 | 1.63E-39 | 2.80E-37 |
| THBS4 | ILMN_1736078 | 1.32 | 3.65E-12 | 6.73E-11 |
| COL8A1 | ILMN_2402392 | 1.32 | 2.40E-27 | 1.81E-25 |
| ISG15 | ILMN_2054019 | 1.32 | 2.14E-34 | 2.60E-32 |
| COL10A1 | ILMN_1672776 | 1.3 | 1.03E-20 | 4.63E-19 |
| TFF3 | ILMN_1811387 | 1.3 | 3.35E-11 | 5.46E-10 |
| MMP9 | ILMN_1796316 | 1.27 | 5.94E-21 | 2.72E-19 |
| UBE2C | ILMN_2301083 | 1.26 | 3.37E-26 | 2.38E-24 |
| GPNMB | ILMN_1801205 | 1.26 | 1.14E-26 | 8.36E-25 |
| OLFML2B | ILMN_1765557 | 1.26 | 4.86E-43 | 1.03E-40 |
| PDGFRB | ILMN_1815057 | 1.25 | 3.43E-37 | 5.00E-35 |
| COL7A1 | ILMN_1751161 | 1.24 | 8.12E-24 | 4.72E-22 |
| KRT6B | ILMN_1721354 | 1.23 | 4.96E-10 | 6.99E-09 |
| TNFRSF6B | ILMN_2331231 | 1.22 | 8.26E-17 | 2.53E-15 |
| APOE | ILMN_1740938 | 1.21 | 9.13E-24 | 5.27E-22 |
| MGC4677 | ILMN_2143795 | 1.21 | 2.19E-48 | 6.28E-46 |
| OLR1 | ILMN_1723035 | 1.2 | 3.38E-28 | 2.70E-26 |
| UBE2C | ILMN_1714730 | 1.19 | 1.11E-24 | 6.80E-23 |
| RARRES1 | ILMN_1743620 | 1.19 | 8.89E-23 | 4.66E-21 |
| DMBT1 | ILMN_2410612 | 1.18 | 1.03E-05 | 7.74E-05 |
| PLAU | ILMN_1656057 | 1.18 | 1.95E-32 | 2.10E-30 |
| LY6E | ILMN_1695404 | 1.17 | 1.21E-27 | 9.27E-26 |
| SOD2 | ILMN_2336781 | 1.17 | 3.41E-32 | 3.60E-30 |
| S100A10 | ILMN_1796712 | 1.17 | 1.46E-52 | 5.43E-50 |
| PMEPA1 | ILMN_1734276 | 1.16 | 8.85E-30 | 8.06E-28 |
| FOXC1 | ILMN_1738401 | 1.15 | 3.43E-23 | 1.87E-21 |
| IGFBP7 | ILMN_2062468 | 1.13 | 3.81E-26 | 2.66E-24 |
| COL11A1 | ILMN_1789507 | 1.13 | 1.83E-14 | 4.39E-13 |
| TNFRSF12A | ILMN_1689004 | 1.13 | 2.99E-29 | 2.61E-27 |
| THBS1 | ILMN_1686116 | 1.12 | 3.89E-21 | 1.80E-19 |
| S100A10 | ILMN_2046730 | 1.11 | 5.40E-56 | 2.39E-53 |
| CLDN3 | ILMN_1723042 | 1.11 | 1.57E-12 | 3.03E-11 |
| RAB31 | ILMN_1660691 | 1.11 | 2.11E-34 | 2.58E-32 |
| IFITM3 | ILMN_1805750 | 1.11 | 5.26E-44 | 1.19E-41 |
| IGF2BP3 | ILMN_1807423 | 1.11 | 4.95E-16 | 1.41E-14 |
| GREM1 | ILMN_1752965 | 1.11 | 2.55E-14 | 6.04E-13 |
| PLA2G7 | ILMN_1701195 | 1.11 | 1.84E-30 | 1.75E-28 |
| DAP3 | ILMN_1781680 | 1.1 | 2.72E-70 | 2.90E-67 |
| OLFM4 | ILMN_1753954 | 1.1 | 6.66E-08 | 7.09E-07 |
| HEYL | ILMN_1654324 | 1.09 | 1.67E-28 | 1.37E-26 |
| FSCN1 | ILMN_1808707 | 1.09 | 2.52E-31 | 2.52E-29 |
| HOXC6 | ILMN_1794492 | 1.08 | 6.99E-23 | 3.70E-21 |
| REG4 | ILMN_2169383 | 1.07 | 3.29E-05 | 2.26E-04 |
| HKDC1 | ILMN_1752502 | 1.07 | 3.49E-16 | 1.01E-14 |
| CDH3 | ILMN_1704294 | 1.07 | 8.60E-21 | 3.87E-19 |
| CLDN2 | ILMN_1795190 | 1.07 | 4.61E-11 | 7.35E-10 |
| HOXB7 | ILMN_1702125 | 1.06 | 1.39E-25 | 9.19E-24 |
| CDH11 | ILMN_1672611 | 1.06 | 7.39E-27 | 5.45E-25 |
| SERPINE2 | ILMN_1655595 | 1.05 | 1.71E-21 | 8.20E-20 |
| C10ORF10 | ILMN_1767556 | 1.05 | 1.59E-22 | 8.15E-21 |
| TGFBI | ILMN_1663866 | 1.05 | 4.90E-36 | 6.67E-34 |
| CXCL9 | ILMN_1745356 | 1.05 | 1.25E-12 | 2.44E-11 |
| MUC17 | ILMN_1724375 | 1.05 | 1.09E-06 | 9.71E-06 |
| CTSK | ILMN_1758895 | 1.05 | 7.60E-23 | 4.00E-21 |
| MMP12 | ILMN_2073758 | 1.05 | 7.26E-11 | 1.13E-09 |
| SERPINH1 | ILMN_1751028 | 1.05 | 5.25E-44 | 1.19E-41 |
| CXCL10 | ILMN_1791759 | 1.05 | 4.58E-13 | 9.42E-12 |
| TOP2A | ILMN_1686097 | 1.03 | 1.14E-20 | 5.07E-19 |
| SHFM1 | ILMN_2128128 | 1.03 | 4.90E-63 | 3.21E-60 |
| CLRN3 | ILMN_2067408 | 1.03 | 6.88E-11 | 1.07E-09 |
| LUM | ILMN_1790529 | 1.03 | 2.35E-17 | 7.55E-16 |
| SERPINE1 | ILMN_1744381 | 1.03 | 5.80E-25 | 3.64E-23 |
| GDF15 | ILMN_2188862 | 1.02 | 1.51E-14 | 3.66E-13 |
| HAVCR2 | ILMN_1693826 | 1.02 | 3.92E-33 | 4.38E-31 |
| GPNMB | ILMN_2407389 | 1.01 | 1.22E-23 | 6.97E-22 |
| TUBB3 | ILMN_1791726 | 1.01 | 3.41E-26 | 2.40E-24 |
| COL18A1 | ILMN_1806733 | 1.01 | 8.24E-34 | 9.82E-32 |
| SFRP2 | ILMN_1722898 | 1.01 | 6.15E-09 | 7.55E-08 |
| CTSL1 | ILMN_2374036 | 1.01 | 1.01E-32 | 1.11E-30 |
| CTSL1 | ILMN_1812995 | 1 | 1.31E-30 | 1.25E-28 |
| TNFAIP6 | ILMN_1785732 | 1 | 3.98E-21 | 1.84E-19 |
| FCER1G | ILMN_2123743 | 1 | 4.40E-24 | 2.60E-22 |
| UBE2I | ILMN_1810474 | 1 | 6.51E-64 | 4.50E-61 |
| CXCL14 | ILMN_1748323 | -1 | 1.94E-16 | 5.74E-15 |
| ARSD | ILMN_1684873 | -1 | 2.73E-39 | 4.56E-37 |
| IL1R2 | ILMN_1772131 | -1 | 9.29E-16 | 2.56E-14 |
| CYB5R1 | ILMN_1729237 | -1 | 2.17E-52 | 7.99E-50 |
| CMBL | ILMN_1709634 | -1 | 1.29E-11 | 2.21E-10 |
| GAMT | ILMN_1756469 | -1 | 4.72E-26 | 3.25E-24 |
| CMTM4 | ILMN_1762718 | -1 | 8.41E-43 | 1.72E-40 |
| ANKRD22 | ILMN_1799848 | -1 | 1.44E-20 | 6.35E-19 |
| EYA2 | ILMN_2322996 | -1.01 | 6.33E-16 | 1.77E-14 |
| FAM107A | ILMN_1743445 | -1.01 | 3.90E-24 | 2.31E-22 |
| CFD | ILMN_1777190 | -1.01 | 3.85E-14 | 8.92E-13 |
| CYB5A | ILMN_2312194 | -1.01 | 2.32E-33 | 2.64E-31 |
| ACACB | ILMN_1763852 | -1.01 | 7.48E-31 | 7.27E-29 |
| SLC2A12 | ILMN_1766261 | -1.01 | 1.12E-37 | 1.67E-35 |
| DHRS7 | ILMN_1807455 | -1.01 | 2.05E-51 | 7.20E-49 |
| HPN | ILMN_1687235 | -1.01 | 2.25E-29 | 2.00E-27 |
| LTB4DH | ILMN_1704531 | -1.02 | 1.37E-30 | 1.30E-28 |
| C9ORF61 | ILMN_2179717 | -1.02 | 8.62E-25 | 5.32E-23 |
| KLF4 | ILMN_2137789 | -1.02 | 4.19E-22 | 2.11E-20 |
| C10ORF116 | ILMN_1680110 | -1.02 | 2.97E-16 | 8.58E-15 |
| UGT2B17 | ILMN_1752214 | -1.02 | 4.92E-30 | 4.57E-28 |
| RPESP | ILMN_1808245 | -1.02 | 1.39E-13 | 3.04E-12 |
| RPS4Y1 | ILMN_1783142 | -1.03 | 1.93E-05 | 1.38E-04 |
| ISL1 | ILMN_1763390 | -1.03 | 7.12E-21 | 3.22E-19 |
| GPR64 | ILMN_2349071 | -1.03 | 7.34E-32 | 7.62E-30 |
| TESC | ILMN_1750181 | -1.03 | 1.84E-12 | 3.52E-11 |
| FBXL13 | ILMN_1791253 | -1.03 | 3.15E-53 | 1.20E-50 |
| FMO5 | ILMN_1811632 | -1.03 | 5.78E-27 | 4.28E-25 |
| TRIM74 | ILMN_2172547 | -1.03 | 7.07E-63 | 4.56E-60 |
| DNER | ILMN_1791679 | -1.04 | 1.18E-39 | 2.05E-37 |
| AFAR3 | ILMN_1702696 | -1.04 | 3.10E-38 | 4.76E-36 |
| NEDD4L | ILMN_1733627 | -1.04 | 1.10E-50 | 3.71E-48 |
| SPINK1 | ILMN_1787266 | -1.05 | 2.12E-08 | 2.43E-07 |
| HS.155736 | ILMN_1887357 | -1.05 | 2.63E-22 | 1.34E-20 |
| RAB27A | ILMN_1665859 | -1.05 | 2.19E-40 | 3.94E-38 |
| PPFIBP2 | ILMN_1675656 | -1.05 | 6.81E-49 | 2.02E-46 |
| MUC6 | ILMN_1683416 | -1.05 | 7.20E-56 | 3.11E-53 |
| SYTL2 | ILMN_2336609 | -1.05 | 9.61E-25 | 5.89E-23 |
| CYP4X1 | ILMN_2175317 | -1.05 | 2.63E-17 | 8.39E-16 |
| ABCC5 | ILMN_1651964 | -1.05 | 2.47E-52 | 9.00E-50 |
| CLDN18 | ILMN_1662484 | -1.06 | 1.52E-23 | 8.61E-22 |
| STARD10 | ILMN_1717052 | -1.06 | 5.38E-28 | 4.22E-26 |
| TMEM171 | ILMN_1728009 | -1.06 | 2.97E-33 | 3.36E-31 |
| COBLL1 | ILMN_1761260 | -1.06 | 1.42E-46 | 3.71E-44 |
| LOC647450 | ILMN_1699214 | -1.06 | 2.80E-08 | 3.14E-07 |
| DGKD | ILMN_1765326 | -1.06 | 1.82E-53 | 7.14E-51 |
| LOC653344 | ILMN_1693836 | -1.06 | 1.72E-39 | 2.94E-37 |
| CIDEC | ILMN_2174437 | -1.06 | 4.32E-13 | 8.92E-12 |
| ABCA8 | ILMN_1796801 | -1.06 | 6.03E-20 | 2.53E-18 |
| APOBEC2 | ILMN_1719143 | -1.07 | 1.01E-34 | 1.26E-32 |
| MGC29506 | ILMN_2193233 | -1.07 | 1.54E-15 | 4.16E-14 |
| RPRM | ILMN_1665425 | -1.07 | 2.90E-32 | 3.07E-30 |
| C11ORF9 | ILMN_1811437 | -1.07 | 2.66E-43 | 5.70E-41 |
| FGA | ILMN_2381945 | -1.07 | 3.57E-30 | 3.33E-28 |
| LOC400451 | ILMN_1652797 | -1.07 | 2.16E-32 | 2.32E-30 |
| TM7SF2 | ILMN_1690040 | -1.07 | 4.56E-41 | 8.46E-39 |
| LOC650200 | ILMN_1741597 | -1.07 | 2.06E-33 | 2.37E-31 |
| RDH12 | ILMN_1792182 | -1.08 | 3.53E-56 | 1.58E-53 |
| IQGAP2 | ILMN_1769433 | -1.09 | 1.91E-33 | 2.20E-31 |
| TMEM30B | ILMN_1752935 | -1.09 | 3.00E-35 | 3.88E-33 |
| APM-1 | ILMN_1796737 | -1.09 | 9.03E-35 | 1.14E-32 |
| LOC388610 | ILMN_1695946 | -1.09 | 2.20E-16 | 6.46E-15 |
| C7ORF41 | ILMN_1672605 | -1.09 | 1.06E-32 | 1.16E-30 |
| ZNF533 | ILMN_1657087 | -1.09 | 3.63E-41 | 6.78E-39 |
| FBP1 | ILMN_1728799 | -1.09 | 6.35E-25 | 3.97E-23 |
| MAMDC2 | ILMN_1679391 | -1.1 | 2.00E-19 | 7.92E-18 |
| FOXA1 | ILMN_1766650 | -1.1 | 6.81E-18 | 2.32E-16 |
| LOC652775 | ILMN_1695891 | -1.1 | 5.31E-12 | 9.56E-11 |
| FOLR1 | ILMN_2346339 | -1.1 | 1.24E-19 | 5.00E-18 |
| C20ORF114 | ILMN_1733904 | -1.11 | 5.02E-06 | 4.00E-05 |
| C6ORF85 | ILMN_1705116 | -1.11 | 4.07E-39 | 6.70E-37 |
| RAB17 | ILMN_2052373 | -1.12 | 2.72E-31 | 2.70E-29 |
| C21ORF51 | ILMN_1777221 | -1.12 | 6.19E-72 | 7.39E-69 |
| ECHDC3 | ILMN_2072178 | -1.12 | 1.55E-19 | 6.16E-18 |
| SCNN1G | ILMN_2153837 | -1.12 | 3.18E-66 | 2.61E-63 |
| SH3BGRL2 | ILMN_1762764 | -1.13 | 1.18E-43 | 2.60E-41 |
| PLLP | ILMN_1787673 | -1.13 | 2.31E-29 | 2.04E-27 |
| FOXQ1 | ILMN_1669046 | -1.13 | 1.44E-17 | 4.75E-16 |
| SLC28A2 | ILMN_1801886 | -1.13 | 7.25E-12 | 1.28E-10 |
| GPX3 | ILMN_1726666 | -1.13 | 1.97E-35 | 2.59E-33 |
| CYP2S1 | ILMN_1705403 | -1.14 | 4.77E-23 | 2.56E-21 |
| TNFRSF17 | ILMN_1768016 | -1.14 | 1.25E-25 | 8.30E-24 |
| GPRC5C | ILMN_1724211 | -1.14 | 7.71E-44 | 1.73E-41 |
| ABCC5 | ILMN_1706531 | -1.14 | 2.43E-45 | 5.82E-43 |
| OSBPL7 | ILMN_1720865 | -1.14 | 3.91E-37 | 5.65E-35 |
| PLA2G10 | ILMN_1762561 | -1.14 | 2.52E-12 | 4.74E-11 |
| PMS2L1 | ILMN_1752028 | -1.14 | 1.01E-57 | 5.10E-55 |
| DEFA5 | ILMN_1770424 | -1.15 | 4.28E-08 | 4.69E-07 |
| SCIN | ILMN_1813561 | -1.15 | 1.29E-26 | 9.41E-25 |
| CYP4F12 | ILMN_1809384 | -1.15 | 1.64E-23 | 9.23E-22 |
| GSTA2 | ILMN_2168747 | -1.15 | 6.37E-23 | 3.39E-21 |
| CMTM4 | ILMN_1815319 | -1.16 | 2.59E-50 | 8.34E-48 |
| PRDM16 | ILMN_2343264 | -1.16 | 2.22E-46 | 5.70E-44 |
| RNASE4 | ILMN_1776602 | -1.16 | 5.84E-33 | 6.49E-31 |
| ST6GALNAC1 | ILMN_1772951 | -1.16 | 1.55E-20 | 6.78E-19 |
| FOXA3 | ILMN_1792733 | -1.16 | 3.28E-27 | 2.46E-25 |
| IRX2 | ILMN_1782412 | -1.16 | 1.66E-20 | 7.27E-19 |
| UBL3 | ILMN_1810729 | -1.16 | 3.51E-65 | 2.66E-62 |
| AGXT2L1 | ILMN_1757807 | -1.16 | 3.63E-71 | 4.21E-68 |
| LOC651751 | ILMN_1674228 | -1.17 | 7.49E-11 | 1.16E-09 |
| VILL | ILMN_1778650 | -1.17 | 2.43E-33 | 2.76E-31 |
| FA2H | ILMN_1791531 | -1.17 | 1.68E-41 | 3.18E-39 |
| LTB4DH | ILMN_2225537 | -1.17 | 8.72E-38 | 1.30E-35 |
| PKIB | ILMN_2337263 | -1.17 | 1.65E-34 | 2.05E-32 |
| GPT | ILMN_1795257 | -1.17 | 8.67E-28 | 6.71E-26 |
| C21ORF51 | ILMN_2356311 | -1.17 | 1.11E-66 | 9.50E-64 |
| S100P | ILMN_1801216 | -1.18 | 2.88E-13 | 6.07E-12 |
| GCNT1 | ILMN_1666545 | -1.18 | 3.64E-26 | 2.56E-24 |
| ADA | ILMN_1803686 | -1.18 | 1.55E-40 | 2.81E-38 |
| PLAC8 | ILMN_1653026 | -1.19 | 1.62E-15 | 4.36E-14 |
| LOC643847 | ILMN_1666985 | -1.19 | 3.60E-92 | 1.01E-88 |
| GATA5 | ILMN_1760153 | -1.19 | 8.83E-25 | 5.43E-23 |
| ARL14 | ILMN_2232463 | -1.19 | 9.09E-20 | 3.72E-18 |
| SLC7A8 | ILMN_1807894 | -1.19 | 9.08E-51 | 3.08E-48 |
| PLAC8 | ILMN_2093343 | -1.2 | 5.29E-15 | 1.35E-13 |
| AADAC | ILMN_1760414 | -1.2 | 1.50E-18 | 5.45E-17 |
| SLC9A1 | ILMN_1800425 | -1.21 | 5.30E-45 | 1.26E-42 |
| C1ORF116 | ILMN_1706483 | -1.21 | 1.13E-28 | 9.33E-27 |
| DAZ4 | ILMN_2404951 | -1.21 | 7.94E-13 | 1.59E-11 |
| SLC25A4 | ILMN_1681670 | -1.22 | 1.23E-38 | 1.98E-36 |
| LOC650867 | ILMN_1753686 | -1.22 | 2.11E-13 | 4.53E-12 |
| C1ORF116 | ILMN_2073446 | -1.22 | 9.97E-31 | 9.64E-29 |
| C9ORF152 | ILMN_1781745 | -1.22 | 3.72E-19 | 1.43E-17 |
| PXMP2 | ILMN_1681634 | -1.22 | 1.18E-54 | 4.79E-52 |
| FUT9 | ILMN_1878007 | -1.23 | 1.19E-66 | 9.99E-64 |
| DUOX1 | ILMN_1690289 | -1.23 | 2.67E-50 | 8.54E-48 |
| DPT | ILMN_1708107 | -1.23 | 3.91E-28 | 3.12E-26 |
| ANG | ILMN_1696974 | -1.23 | 2.25E-35 | 2.93E-33 |
| POU2AF1 | ILMN_1811049 | -1.23 | 6.64E-41 | 1.21E-38 |
| LTF | ILMN_1677920 | -1.24 | 6.65E-21 | 3.02E-19 |
| C20ORF127 | ILMN_1662640 | -1.24 | 3.46E-27 | 2.59E-25 |
| RNASE4 | ILMN_2294978 | -1.24 | 6.07E-36 | 8.21E-34 |
| RNASE4 | ILMN_2294976 | -1.24 | 3.31E-38 | 5.07E-36 |
| APLP1 | ILMN_1763834 | -1.25 | 1.74E-64 | 1.25E-61 |
| CLCNKA | ILMN_1787576 | -1.25 | 4.62E-46 | 1.16E-43 |
| ITPKA | ILMN_1776516 | -1.25 | 1.73E-28 | 1.42E-26 |
| FCGBP | ILMN_1718984 | -1.25 | 1.23E-18 | 4.50E-17 |
| PLLP | ILMN_2082865 | -1.26 | 9.90E-30 | 8.91E-28 |
| SULT2A1 | ILMN_1692983 | -1.26 | 2.78E-42 | 5.43E-40 |
| ALDOB | ILMN_1747716 | -1.26 | 1.19E-10 | 1.82E-09 |
| ALDH6A1 | ILMN_1785284 | -1.27 | 3.36E-83 | 6.97E-80 |
| SELENBP1 | ILMN_1680652 | -1.27 | 7.49E-46 | 1.84E-43 |
| CGNL1 | ILMN_1730229 | -1.28 | 5.11E-28 | 4.04E-26 |
| ANG | ILMN_1760727 | -1.28 | 4.15E-39 | 6.80E-37 |
| TCEA3 | ILMN_1726928 | -1.28 | 4.93E-29 | 4.18E-27 |
| UGT2B15 | ILMN_2048414 | -1.29 | 1.62E-38 | 2.54E-36 |
| MAOA | ILMN_1663640 | -1.3 | 2.37E-26 | 1.69E-24 |
| DAZ4 | ILMN_1754528 | -1.3 | 6.16E-14 | 1.40E-12 |
| IGFBP2 | ILMN_1725193 | -1.31 | 9.73E-24 | 5.60E-22 |
| FBP2 | ILMN_1804005 | -1.31 | 1.25E-65 | 9.61E-63 |
| AGR2 | ILMN_1814151 | -1.31 | 1.15E-18 | 4.24E-17 |
| ORM2 | ILMN_1731785 | -1.31 | 9.03E-24 | 5.22E-22 |
| ALDH1A1 | ILMN_2096372 | -1.33 | 2.43E-24 | 1.45E-22 |
| PROM2 | ILMN_1761946 | -1.33 | 2.83E-21 | 1.33E-19 |
| LOC388743 | ILMN_1753766 | -1.34 | 2.37E-26 | 1.69E-24 |
| SLC25A4 | ILMN_2211780 | -1.34 | 4.26E-42 | 8.26E-40 |
| ADHFE1 | ILMN_1702858 | -1.35 | 8.88E-64 | 6.02E-61 |
| MT1A | ILMN_1691156 | -1.35 | 1.18E-39 | 2.06E-37 |
| CLCNKA | ILMN_2364072 | -1.35 | 2.06E-50 | 6.71E-48 |
| KRT20 | ILMN_1794729 | -1.36 | 9.06E-14 | 2.02E-12 |
| SIDT2 | ILMN_1791912 | -1.36 | 6.40E-93 | 2.10E-89 |
| LOC643959 | ILMN_1759708 | -1.37 | 3.91E-51 | 1.35E-48 |
| SCNN1A | ILMN_1713995 | -1.37 | 1.74E-18 | 6.27E-17 |
| PXMP2 | ILMN_1799015 | -1.37 | 2.87E-61 | 1.73E-58 |
| CYP3A5 | ILMN_1810942 | -1.38 | 1.06E-20 | 4.73E-19 |
| EEF1A2 | ILMN_2108735 | -1.38 | 2.75E-22 | 1.39E-20 |
| ADH7 | ILMN_2223359 | -1.39 | 1.19E-72 | 1.46E-69 |
| XYLT2 | ILMN_1799815 | -1.39 | 2.30E-76 | 3.62E-73 |
| FOXA2 | ILMN_1668052 | -1.39 | 7.96E-21 | 3.59E-19 |
| NQO1 | ILMN_1720282 | -1.39 | 1.10E-32 | 1.20E-30 |
| HS.25318 | ILMN_1837428 | -1.4 | 1.21E-30 | 1.16E-28 |
| HS.481464 | ILMN_1836218 | -1.4 | 4.60E-26 | 3.19E-24 |
| HMGCS2 | ILMN_1815203 | -1.41 | 1.78E-11 | 3.01E-10 |
| AQP4 | ILMN_1747683 | -1.41 | 8.92E-83 | 1.67E-79 |
| VSIG1 | ILMN_1806198 | -1.42 | 1.09E-19 | 4.43E-18 |
| MT1X | ILMN_1775170 | -1.42 | 4.67E-38 | 7.01E-36 |
| SH3GL2 | ILMN_1661491 | -1.42 | 1.42E-70 | 1.55E-67 |
| CTSE | ILMN_1693738 | -1.42 | 5.03E-23 | 2.69E-21 |
| HS.19193 | ILMN_1831106 | -1.43 | 9.46E-30 | 8.55E-28 |
| PIGR | ILMN_1685387 | -1.43 | 3.77E-11 | 6.10E-10 |
| SCNN1B | ILMN_1740917 | -1.43 | 4.10E-91 | 1.01E-87 |
| CKM | ILMN_1757521 | -1.44 | 4.48E-73 | 5.69E-70 |
| MFSD4 | ILMN_1729734 | -1.44 | 4.55E-87 | 1.05E-83 |
| AKR1C3 | ILMN_1713124 | -1.44 | 2.95E-26 | 2.09E-24 |
| MSMB | ILMN_1699243 | -1.45 | 8.05E-10 | 1.10E-08 |
| MTE | ILMN_2136089 | -1.45 | 6.09E-39 | 9.89E-37 |
| MAL | ILMN_2320330 | -1.47 | 2.88E-58 | 1.55E-55 |
| MT1E | ILMN_2173611 | -1.48 | 1.69E-31 | 1.72E-29 |
| TMPRSS2 | ILMN_1791123 | -1.48 | 2.24E-33 | 2.57E-31 |
| PNPLA7 | ILMN_1662587 | -1.49 | 4.34E-60 | 2.51E-57 |
| AKR7A3 | ILMN_1718483 | -1.49 | 1.69E-42 | 3.38E-40 |
| P2RXL1 | ILMN_1716335 | -1.5 | 3.31E-69 | 3.18E-66 |
| FAM46C | ILMN_1713266 | -1.51 | 5.77E-43 | 1.21E-40 |
| SLC9A4 | ILMN_1701975 | -1.51 | 3.18E-74 | 4.47E-71 |
| MFSD4 | ILMN_2209238 | -1.52 | 1.65E-85 | 3.61E-82 |
| SULT1C2 | ILMN_1766079 | -1.52 | 3.22E-29 | 2.80E-27 |
| MLPH | ILMN_1795342 | -1.52 | 7.62E-27 | 5.61E-25 |
| SLC9A2 | ILMN_1738849 | -1.53 | 4.97E-48 | 1.40E-45 |
| SLC26A9 | ILMN_1683102 | -1.53 | 2.52E-53 | 9.73E-51 |
| FER1L4 | ILMN_2206722 | -1.54 | 3.67E-34 | 4.43E-32 |
| GPRC5C | ILMN_2352090 | -1.55 | 3.60E-55 | 1.51E-52 |
| LOC130576 | ILMN_1693119 | -1.55 | 3.38E-36 | 4.70E-34 |
| TRIM50 | ILMN_1700408 | -1.55 | 1.50E-91 | 3.92E-88 |
| HOMER2 | ILMN_1671486 | -1.56 | 1.26E-38 | 2.03E-36 |
| RNASE1 | ILMN_1795183 | -1.56 | 5.28E-51 | 1.81E-48 |
| PDIA2 | ILMN_1804444 | -1.58 | 1.12E-41 | 2.14E-39 |
| ALDH1A1 | ILMN_1709348 | -1.58 | 3.85E-29 | 3.30E-27 |
| CKMT2 | ILMN_1764266 | -1.58 | 2.90E-56 | 1.31E-53 |
| AKR1C4 | ILMN_1687757 | -1.59 | 1.11E-31 | 1.14E-29 |
| RNASE1 | ILMN_2333670 | -1.6 | 2.17E-38 | 3.37E-36 |
| HYAL1 | ILMN_1739813 | -1.6 | 9.50E-35 | 1.19E-32 |
| C6ORF105 | ILMN_2078592 | -1.61 | 9.15E-36 | 1.22E-33 |
| GPT2 | ILMN_1684158 | -1.62 | 3.98E-57 | 1.96E-54 |
| LOC284422 | ILMN_1774375 | -1.67 | 1.09E-25 | 7.26E-24 |
| LOC644151 | ILMN_1695397 | -1.68 | 6.71E-28 | 5.23E-26 |
| NKX6-2 | ILMN_1786989 | -1.7 | 5.06E-29 | 4.28E-27 |
| FGA | ILMN_1779017 | -1.71 | 3.15E-50 | 9.98E-48 |
| HDC | ILMN_1792323 | -1.72 | 6.91E-45 | 1.64E-42 |
| UGT2B17 | ILMN_1808677 | -1.73 | 3.78E-38 | 5.74E-36 |
| BCAS1 | ILMN_2136147 | -1.73 | 2.83E-34 | 3.43E-32 |
| MT1F | ILMN_1718766 | -1.74 | 1.58E-48 | 4.58E-46 |
| MUC6 | ILMN_1680070 | -1.74 | 1.14E-61 | 7.02E-59 |
| SULT1C2 | ILMN_2415329 | -1.74 | 1.03E-31 | 1.06E-29 |
| CCKBR | ILMN_1760088 | -1.75 | 1.74E-67 | 1.55E-64 |
| C5ORF32 | ILMN_1761566 | -1.76 | 5.79E-59 | 3.25E-56 |
| CTSE | ILMN_2387224 | -1.76 | 1.38E-23 | 7.83E-22 |
| CYP2C18 | ILMN_1683121 | -1.77 | 1.76E-34 | 2.17E-32 |
| CLDN18 | ILMN_2389054 | -1.78 | 4.29E-17 | 1.34E-15 |
| FGA | ILMN_1656487 | -1.79 | 8.57E-59 | 4.75E-56 |
| BCAS1 | ILMN_1733042 | -1.8 | 1.74E-43 | 3.77E-41 |
| GC | ILMN_1736162 | -1.82 | 2.24E-42 | 4.44E-40 |
| MRGPRD | ILMN_1714980 | -1.82 | 2.25E-62 | 1.41E-59 |
| CLDN18 | ILMN_1696284 | -1.82 | 5.03E-14 | 1.15E-12 |
| RAP1GAP | ILMN_1776519 | -1.83 | 2.51E-40 | 4.48E-38 |
| IRX3 | ILMN_1811468 | -1.83 | 1.19E-43 | 2.60E-41 |
| KIAA1324 | ILMN_1771482 | -1.84 | 2.83E-29 | 2.48E-27 |
| CAPN9 | ILMN_1690923 | -1.85 | 3.52E-48 | 1.00E-45 |
| TCN1 | ILMN_1768469 | -1.86 | 7.22E-18 | 2.45E-16 |
| LOC441282 | ILMN_2278335 | -1.86 | 5.49E-26 | 3.76E-24 |
| CHIA | ILMN_1679647 | -1.86 | 1.77E-75 | 2.67E-72 |
| CAPN13 | ILMN_1677108 | -1.88 | 1.16E-56 | 5.43E-54 |
| FLJ42875 | ILMN_1732000 | -1.9 | 2.01E-131 | 2.63E-127 |
| CHRD | ILMN_1795582 | -1.9 | 1.68E-64 | 1.22E-61 |
| FAM3B | ILMN_2355486 | -1.9 | 2.93E-30 | 2.75E-28 |
| C6ORF58 | ILMN_1754920 | -1.91 | 2.95E-16 | 8.53E-15 |
| CTSE | ILMN_1799887 | -1.91 | 1.44E-23 | 8.18E-22 |
| LOC644844 | ILMN_1661078 | -1.93 | 3.35E-17 | 1.06E-15 |
| ADH1A | ILMN_1764309 | -1.95 | 2.09E-23 | 1.17E-21 |
| REP15 | ILMN_1665884 | -1.96 | 2.56E-56 | 1.17E-53 |
| CXCL17 | ILMN_1796337 | -1.99 | 1.69E-41 | 3.18E-39 |
| KLK11 | ILMN_1695924 | -2 | 5.94E-26 | 4.03E-24 |
| HPGD | ILMN_2166457 | -2.01 | 1.10E-42 | 2.23E-40 |
| GSTA1 | ILMN_1701831 | -2.02 | 1.10E-40 | 2.01E-38 |
| REG3A | ILMN_2382679 | -2.02 | 2.03E-12 | 3.86E-11 |
| TMED6 | ILMN_1748899 | -2.1 | 1.47E-69 | 1.48E-66 |
| AKR7A3 | ILMN_2145396 | -2.1 | 2.79E-69 | 2.74E-66 |
| MAL | ILMN_2327860 | -2.1 | 2.85E-54 | 1.14E-51 |
| DPCR1 | ILMN_1687625 | -2.1 | 3.67E-36 | 5.07E-34 |
| GSTA2 | ILMN_1655613 | -2.11 | 5.27E-39 | 8.60E-37 |
| GPER | ILMN_2384056 | -2.12 | 2.91E-61 | 1.74E-58 |
| MT1M | ILMN_1657435 | -2.12 | 3.85E-47 | 1.07E-44 |
| GPER | ILMN_1795298 | -2.13 | 4.31E-73 | 5.66E-70 |
| LOC643834 | ILMN_1768750 | -2.15 | 5.55E-69 | 5.20E-66 |
| LOC647169 | ILMN_1782937 | -2.15 | 6.48E-36 | 8.67E-34 |
| CKB | ILMN_1671478 | -2.16 | 6.20E-55 | 2.54E-52 |
| MT1H | ILMN_2124802 | -2.19 | 4.17E-37 | 5.99E-35 |
| GSTA1 | ILMN_2113470 | -2.21 | 6.12E-34 | 7.34E-32 |
| IGJ | ILMN_2105441 | -2.23 | 3.03E-31 | 3.00E-29 |
| FCGBP | ILMN_2302757 | -2.24 | 1.26E-26 | 9.20E-25 |
| CA2 | ILMN_1662795 | -2.26 | 8.36E-32 | 8.65E-30 |
| SCGB2A1 | ILMN_1732398 | -2.29 | 3.63E-46 | 9.21E-44 |
| REG1A | ILMN_1802441 | -2.31 | 1.30E-17 | 4.31E-16 |
| MT1G | ILMN_1715401 | -2.32 | 2.77E-39 | 4.61E-37 |
| CA2 | ILMN_2199439 | -2.32 | 3.70E-35 | 4.74E-33 |
| SOX21 | ILMN_1783185 | -2.33 | 9.15E-42 | 1.75E-39 |
| LOC728473 | ILMN_1816925 | -2.33 | 7.38E-81 | 1.26E-77 |
| ADH1C | ILMN_1740717 | -2.35 | 5.19E-29 | 4.38E-27 |
| KCNJ16 | ILMN_1813741 | -2.36 | 2.69E-96 | 1.18E-92 |
| SOSTDC1 | ILMN_1715463 | -2.36 | 2.02E-75 | 2.95E-72 |
| AKR1C2 | ILMN_2412336 | -2.42 | 2.53E-37 | 3.71E-35 |
| CA9 | ILMN_1725139 | -2.45 | 7.08E-47 | 1.91E-44 |
| CHIA | ILMN_1798138 | -2.46 | 5.50E-96 | 2.16E-92 |
| ALDH3A1 | ILMN_1702503 | -2.49 | 6.36E-47 | 1.74E-44 |
| ESRRG | ILMN_1661994 | -2.49 | 1.66E-82 | 2.97E-79 |
| ANXA10 | ILMN_1699421 | -2.5 | 5.62E-24 | 3.30E-22 |
| CAPN9 | ILMN_1731073 | -2.53 | 2.04E-50 | 6.69E-48 |
| SST | ILMN_1812824 | -2.57 | 7.20E-61 | 4.23E-58 |
| CLIC6 | ILMN_1699665 | -2.59 | 4.98E-54 | 1.98E-51 |
| VSIG2 | ILMN_1666536 | -2.74 | 2.28E-42 | 4.51E-40 |
| AKR1B10 | ILMN_1672148 | -2.83 | 1.17E-32 | 1.27E-30 |
| CHGA | ILMN_1669410 | -2.86 | 4.73E-50 | 1.49E-47 |
| TFF1 | ILMN_1722489 | -2.94 | 8.64E-29 | 7.19E-27 |
| CPA2 | ILMN_1807503 | -3 | 1.58E-112 | 1.03E-108 |
| PGC | ILMN_1795484 | -3.02 | 1.87E-23 | 1.05E-21 |
| MUC5AC | ILMN_1748303 | -3.15 | 6.98E-30 | 6.38E-28 |
| PSCA | ILMN_1771538 | -3.15 | 3.76E-58 | 2.00E-55 |
| TFF2 | ILMN_1663919 | -3.18 | 2.66E-29 | 2.34E-27 |
| ATP4B | ILMN_1771680 | -3.24 | 6.86E-138 | 1.35E-133 |
| GHRL | ILMN_1696380 | -3.28 | 7.76E-70 | 8.03E-67 |
| KCNE2 | ILMN_1769143 | -3.31 | 3.35E-93 | 1.20E-89 |
| ATP4B | ILMN_1796405 | -3.86 | 1.31E-138 | 5.16E-134 |
| GKN1 | ILMN_1760103 | -3.97 | 1.54E-38 | 2.45E-36 |
| GKN2 | ILMN_1813688 | -4.47 | 5.61E-50 | 1.73E-47 |
| PGA3 | ILMN_1727539 | -4.62 | 8.04E-57 | 3.81E-54 |
| LIPF | ILMN_1781517 | -4.62 | 5.76E-66 | 4.53E-63 |
| PGA5 | ILMN_1717572 | -4.62 | 6.02E-57 | 2.89E-54 |
| PGA3 | ILMN_1687842 | -4.8 | 3.26E-66 | 2.62E-63 |
| PGA3 | ILMN_1677711 | -4.87 | 5.96E-71 | 6.70E-68 |
| GIF | ILMN_1731547 | -4.99 | 4.72E-104 | 2.65E-100 |
| ATP4A | ILMN_2209417 | -5.02 | 1.41E-125 | 1.11E-121 |
| ATP4B | ILMN_1703787 | -5.08 | 1.53E-127 | 1.50E-123 |
| PGA3 | ILMN_2163324 | -5.15 | 2.11E-92 | 6.39E-89 |
| PGA4 | ILMN_2232630 | -5.17 | 9.63E-97 | 4.74E-93 |
